# Limitations of employing a unified physiological equivalent temperature scale for assessing thermal comfort in Taiwan’s Cwa urban climate

**DOI:** 10.1007/s00484-026-03294-2

**Published:** 2026-08-20

**Authors:** Kang-Ting Tsai, Ning-Shiang Chen, Jia-Yu Jhang

**Affiliations:** 1 Program of Landscape and Recreation, National Chung Hsing University, 145 Xingda Rd, Taichung, 402 Taiwan; 2 Program in Industrial and Smart Technology, National Chung Hsing University, No. 15, Guangming Rd, Nantou City, Nantou County 540 Taiwan

**Keywords:** Outdoor thermal comfort, Human biometeorology, Modified physiological equivalent temperature (mPET), Humid subtropical climate, Taiwan climate zones

## Abstract

Taiwan’s national reference for outdoor thermal comfort assessment originates from a single non-urban site in a Cfa zone, whereas the country’s most heat-vulnerable metropolitan areas lie in the Cwa zone. Conventional thermal comfort evaluations rely on single standardized scales, which may not fully reflect variation across different climates and outdoor contexts. This study employed the modified physiologically equivalent temperature (mPET) thermal index as a thermal comfort indicator and established a regionally adaptive and mPET-based thermal perception scale suitable for Taiwan’s Cwa climate. Field studies were conducted in two representative Cwa sites—Zhongxing New Village and the Central Taiwan Science Park—and encompassed seven outdoor spaces with distinct spatial functions and microclimatic characteristics. Microclimatic measurements, questionnaire surveys, and user count observations were considered to examine the relationships among thermal conditions, thermal sensation, thermal comfort, and spatial behavior. The results indicate that a neutral temperature for these sites is 23.3 °C mPET and that a 90% acceptable thermal comfort range is between 21.4 and 25.8 °C mPET. Behavioral thermal adaptation, including shade-seeking and time-shifting behaviors, was also observed in this study. In addition, contextual comparison with PET thresholds reported for other climate zones indicated the regional specificity of the neutral temperatures and physiologically equivalent temperature classifications derived for Taiwan’s Cwa climate, suggesting that a single physiologically equivalent temperature scale may be insufficient for nationwide evaluations. The study findings highlight a need for climate-sensitive and behaviorally informed thermal comfort assessments. The results offer initial empirical support to aid with climate adaptation strategies and outdoor space planning in humid subtropical urban environments, with further validation across additional Cwa sites warranted.

**Supplementary Information:**

The online version contains supplementary material available at https://doi.org/10.1007/s00484-026-03294-2.

## Introduction

The effects of climate change have been intensifying, with the frequency and severity of extreme summer heat events increasing in subtropical regions including Taiwan, posing growing risks to human health and well-being (Speak and Salbitano 2022). Urban populations are disproportionately exposed to these stresses through the urban heat island effect, making outdoor thermal comfort a critical concern in urban planning and climate adaptation, particularly in densely built metropolitan areas. Perceptions of outdoor thermal conditions influence the willingness of individuals to use outdoor spaces, shape behavioral patterns (Reiter and Herde 2003; Vanos et al. 2012), and affect the vibrancy and efficiency of public space usage (Nikolopoulou et al. 2001; Lin et al. 2013). Although Taiwan spans multiple Köppen-Geiger climate zones due to its topography and latitude, the most urbanized and heat-vulnerable areas are concentrated within the Cwa zone (humid subtropical with dry winter and hot summer) (Beck et al. 2023). Microclimatic conditions in these Cwa urban areas, intensified by urban heat island effects, further differentiate the thermal environment experienced by residents from regional climatological averages.

Physiologically equivalent temperature (PET) is a thermal index that was developed on the basis of the human energy balance model by Höppe (1999). PET is widely used in outdoor thermal comfort assessments (Kumar and Sharma 2020; Potchter et al. 2018; Tapias and Schmitt 2014). Over the past 2 decades, PET has been widely used to evaluate outdoor thermal comfort (Potchter et al. 2022). According to Chen and Matzarakis (2018), PET cannot be effectively employed to assess thermal comfort in hot and humid climates. Accordingly, a modified PET (mPET) was developed for such climates. Lin et al. (2019) demonstrated that mPET is more sensitive than is PET to humidity variations, rendering it more suitable than PET for use in subtropical and humid climates. Recent humid-subtropical applications (Li et al. 2024; He et al. 2025) have further confirmed that PET-based scales require region-specific calibration to reflect local thermal perception accurately.

**Fig. 1:**
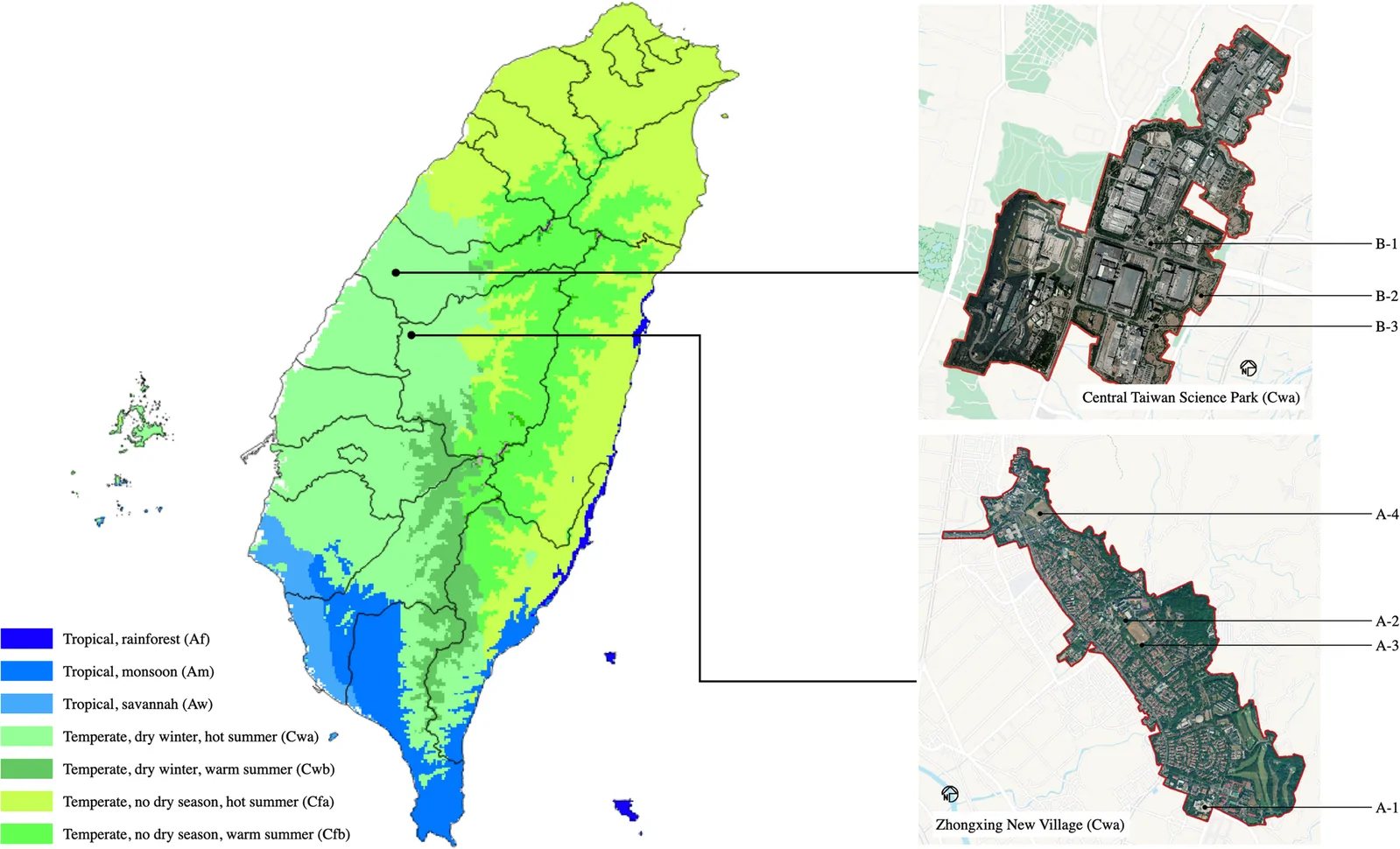
Major Climate Zones in Taiwan and Study Locations. The map illustrates Taiwan’s major climate classifications and highlights the two case study areas: Zhongxing New Village and the Central Taiwan Science Park (CTSP)

The thermal comfort classification currently adopted in Taiwan’s national climate services—including the Central Weather Administration, the Ministry of Health and Welfare, the Ministry of Environment and the TCCIP urban heat hazard indicators (NSTC 2025) is primarily based on the PET scale established by Lin and Matzarakis (2008) at Sun Moon Lake. Under the high-resolution (1 km) Köppen-Geiger classification of Beck et al. (2023), this reference site (23°52′N, 120°55′E; elevation 748 m) lies in a Cfa zone adjacent to Cwa and Cwb classifications. Of the six Köppen zones present in Taiwan, the Cwa zone covers the western lowlands where the largest metropolitan populations and most heat-vulnerable communities are concentrated (Fig. 1). The combined effect of deriving the national reference from a single non-urban site outside the Cwa zone and the well-documented sensitivity of PET-based thermal perception to local climate context (Manavvi and Rajasekar 2022) creates a need to establish a Cwa-specific thermal perception scale grounded in in-situ urban field measurements. Key challenges in applying PET in Taiwan are as follows:

1. Climate-zone alignment: National-level thermal comfort references should ideally be calibrated within, or directly validated for, the climate zone in which they are most heavily applied; the current Cfa-derived scale lacks such direct calibration for the Cwa urban contexts where most policy application occurs.
2. Microclimate variability: Residents in different urban settings within the same Cwa climate zone may exhibit varying thermal expectations and comfort thresholds (Lin 2009; Deng et al. 2022), warranting in-situ field calibration at representative Cwa sites.
3. Urban space interaction: Individuals adapt to outdoor thermal environments through behavioral and positional adjustments, which interact with spatial design and complicate evaluations of comfort levels (Aghamolaei et al. 2022; Chen and Ng 2012; Elnabawi and Hamza 2020; Kleerekoper et al. 2012; Lin et al. 2013).

Building on these considerations, this study tests two hypotheses. First, because the Cwa zone differs from the Cfa zone in seasonal temperature regime and the Cwa urban context differs further through urban heat island intensification, users in Cwa urban environments are expected to exhibit a thermal perception scale—including neutral temperature and acceptable comfort range—that diverges from the existing Taiwan reference derived at the Cfa site. Second, because the existing Taiwan reference was established at a recreational lakeside destination whereas the present sites are everyday urban environments, differences in user activities, behavioral adaptation patterns, and exposure duration are expected to further shape the subjective thermal response and contribute to deviations from the existing reference scale.

This study selected two urban sites with different land-use characteristics—Zhongxing New Village, Nantou, and the Central Taiwan Science Park, Taichung—and conducted microclimatic measurements, nonparticipant behavioral observations, and subjective thermal comfort surveys. These two Cwa sites represent two prevailing modes of frontier urban development around science parks in Taiwan: a rural-planned settlement adjacent to a science-park district, and an urban-planned high-density science-park district. New science-park areas continue to be commissioned and expanded through 2026, underscoring the practical relevance of establishing in-situ thermal benchmarks for such Cwa urban environments. The findings of this study may clarify the applicability and limitations of current PET classification standards within Taiwan’s Cwa urban environments, providing initial empirical evidence that can inform future revisions of climate-zone-specific thermal comfort references and contextualize outdoor space assessment in metropolitan settings.

## Methodology

### Study areas

Both study areas are located within the Cwa climate zone of central Taiwan. They were selected to capture representative urban outdoor environments across the two prevailing modes of frontier urban development around science parks in Taiwan—a rural-adjacent, low-density planned settlement and an urban-adjacent, high-density planned science-park district—thereby providing a broader sampling basis for establishing a Cwa-specific thermal perception scale. Science parks constitute one of Taiwan’s most active frontiers of urban development, with new park areas continuing to be commissioned and expanded through 2026, which underscores the practical relevance of in-situ thermal benchmarking in such Cwa settings.

Zhongxing New Village is located at 23.95° N, 120.68° E, has an elevation of approximately 97 m, and has a total area of about 224 hectares. The average seasonal temperatures in Zhongxing New Village (based on the 2018–2022 records of the nearby Tsaotun station, C0H960, Central Weather Administration) are 18.5 °C (relative humidity ≈ 80%) in the cool season and 26.2 °C (≈ 79%) in the hot season. Zhongxing New Village was established in 1957 as a postwar relocation town and was planned on the basis of Howard’s Garden City theory (Farr 2011; Sharifi 2016) with a focus on administrative and residential functions. The area has high greenery coverage and a comfortable outdoor environment and borders the Central Taiwan Science Park’s Chung Hsing Park area, representing the rural-adjacent science-park development mode. The Central Taiwan Science Park in Taichung is located at 24.21° N, 120.62° E, has an elevation of approximately 149 m, and has a total area of about 454 hectares. The average seasonal temperatures in this park (based on the 2018–2022 records of the nearby Daya–CTSP station, C0F9 × 0) are 17.0 °C (relative humidity ≈ 81%) in the cool season and 25.2 °C (≈ 78%) in the hot season. The Central Taiwan Science Park was established in 2003 as a high-density, urban-adjacent science-park district characterized by extensive impervious paving, large-footprint industrial and office buildings, and limited canopy coverage. The park has a working population exceeding 50,000 and is a major hub for Taiwan’s technology industry.

In both study areas, sites with high usage frequency and high concentrations of individuals were selected for microclimatic monitoring and survey administration. A total of seven measurement sites were identified (Table 1). Both areas share three matched representative urban outdoor space typologies—plaza, playground, and park—that the sampled spaces span the common outdoor settings found in Taiwanese urban areas. An additional open-lawn site (A-4) was included in Zhongxing New Village to reflect its Garden City planning heritage, which features extensive open green spaces not present in the high-density Central Taiwan Science Park. Each site has distinct physical characteristics, spatial typologies and user activities. Following the method of Jin et al. (2018), a 50-meter radius buffer was established around each site as the primary microclimatic influence zone.

Whereas many outdoor thermal comfort studies have focused solely on the hot season, the current research was conducted in both the hot and cool seasons (Lai et al. 2019; Lin et al. 2019). The cool season was defined as December to February, and the hot season was defined as March to November, consistent with the literature (Lin 2009; Lin et al. 2013). Based on the five-year (2018–2022) records of the nearby Tsaotun (station C0H960) and Daya–CTSP (station C0F9 × 0) weather stations operated by Taiwan’s Central Weather Administration, Zhongxing New Village (served by Tsaotun) had cool-season and hot-season mean temperatures of 18.5 °C and 26.2 °C with mean relative humidity of approximately 80% and 79%, respectively, while the Central Taiwan Science Park (served by Daya–CTSP) had cool-season and hot-season mean temperatures of 17.0 °C and 25.2 °C with mean relative humidity of approximately 81% and 78%, respectively. These values illustrate the modest seasonal temperature contrast and the consistently high humidity characteristic of the Cwa climate.

To capture user-relevant data, microclimatic measurements and questionnaire surveys were conducted between 9:00 a.m. and 5:00 p.m., the period during which individuals are most likely to engage in outdoor activities.

**Table 1:**
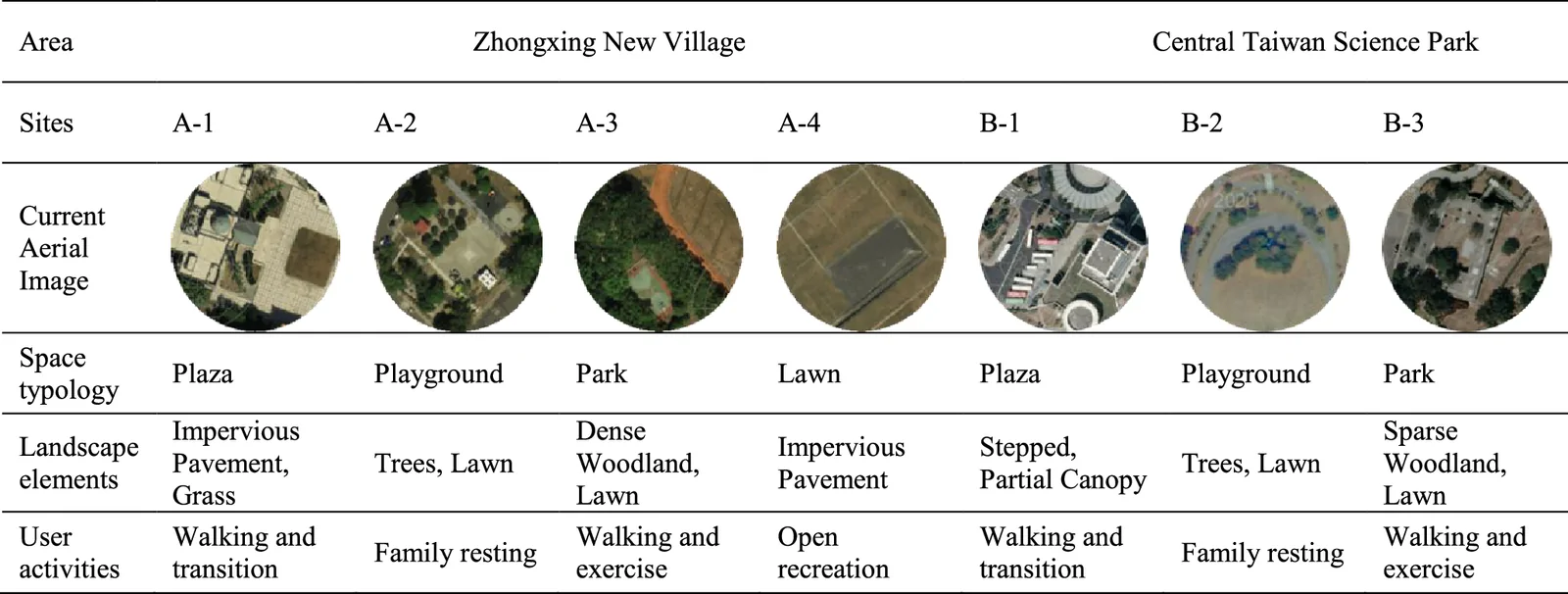
Study investigation sites and description

### Microclimatic measurements

Fieldwork was conducted in 2023, with each of the seven sites measured once per season (cool and hot), yielding 14 site-season measurement campaigns. The schedule of measurement dates, days of the week, corresponding weather stations, and daily meteorological conditions is provided in Table 2. The study employed an integrated approach combining microclimatic measurements, questionnaires, and nonparticipant observations to comprehensively examine users’ thermal perceptions, behavioral responses, and spatial usage patterns across different outdoor environments.

In accordance with ISO 7726 (1998), instruments were mounted on tripods at a height of 1.1 m, which corresponds to the average center of gravity of a standing human body and the standard height of human thermal reception. Instruments were placed at the geometric center of each measurement site, with minor on-site adjustments made to ensure unobstructed exposure to representative microclimatic conditions of the site. Air temperature, globe temperature, and relative humidity were measured using a Tenmars TM-188D WBGT meter (Ta: 0–50 °C, ± 0.8 °C; Tg: 0–80 °C, ± 0.6 °C; RH: 1–99%, ± 3% at 20–80% RH). Wind speed was measured using a Lutron AM-4257SD cup anemometer (0.9–35.0 m/s, ±(2%+0.2 m/s). The sun-exposure status of the instrument station (full sun, partial shade, or full shade) was recorded by field surveyors at each measurement session; this status was also recorded for each respondent in the questionnaire to enable later analysis of behavioral positioning under different radiative conditions (see Sect. 2.4). A summary of the measurements is presented in Table 2.

**Table 2:** Weather stations and microclimate measurement data for each study site

season	Sites	Date	Day	Question- naires	Ta (°C)	Rh (%)	V (m/s)	Precip (mm)	Ta (°C)	Rh (%)	V (m/s)	Tmrt (°C)
					Weather station data				Microclimatic data			
Cool season	A-1	2023/2/11	Sat	22	23.9	66.1	1.1	0.0	27.7	51.1	0.2	32.6
	A-2	2023/1/8	Sun	29	23.3	61	0.6	0.0	26.3	49.9	1.1	38.2
	A-3	2023/1/7	Sat	29	21.7	59.7	0.5	0.0	23.3	54.3	0.8	24.4
	A-4	2023/1/15	Sun	27	22.3	62.4	0.9	0.0	26.6	48.3	2.6	34.9
	B-1	2023/2/10	Fri	35	23.7	72.0	2.3	0.0	24.9	64.2	2.0	27.6
	B-2	2023/1/8	Sun	52	22.4	68.1	1.7	0.0	24.0	57.6	1.5	26.7
	B-3	2023/1/7	Sat	34	21.4	59.4	4.2	0.0	24.1	45.9	1.1	28.9
Hot season	A-1	2023/4/15	Sat	28	28.8	57.6	0.6	0.0	31.5	43.7	1.1	40.6
	A-2	2023/4/16	Sun	26	29.4	53.4	1.2	0.0	31.2	46.4	1.3	35.3
	A-3	2023/4/16	Sun	27	29.4	53.4	1.2	0.0	30.0	48.5	1.4	31.8
	A-4	2023/4/23	Sun	25	28.2	63.4	1.0	0.0	30.9	54.3	2.1	40.0
	B-1	2023/4/18	Tue	24	28.4	66.9	1.5	0.0	28.7	55.3	1.0	31.3
	B-2	2023/4/16	Sun	30	28.3	62.1	2.2	0.0	29.3	49.2	1.8	33.0
	B-3	2023/4/16	Sun	25	28.3	62.1	2.2	0.0	31.7	45.9	1.3	39.4

*Weather-station data source: Central Weather Administration, Taiwan. Tsaotun station (23°59′N, 120°41′E) serves Zhongxing New Village (A1-A4); Daya (CTSP) station (24°14′N, 120°37′E) serves the Central Taiwan Science Park (B1-B3)

### Nonparticipant observation

To analyze the relationship between thermal comfort and public space usage, this study conducted systematic observation of nonparticipants (i.e., the study counted the number of individuals at each site at specific time points) (Sharmin et al. 2019). Observations were synchronized with the microclimatic measurements and questionnaire results. In line with the literature (Lin et al. 2013; Deng et al. 2022), user counts were conducted every 30 min on the hour and half-hour. Observation radii were set at 50 m from the center of each measurement site, and 16 observation sessions were conducted per site per day.

Behavioral and positional thermal adaptation was estimated through two complementary approaches: (a) variations in user counts at each site under different mPET conditions, obtained from the nonparticipant observations described above; and (b) the sun-exposure status (full sun, partial shade, or full shade) of each respondent at the moment of questionnaire completion, recorded in parallel with the microclimatic measurements (see Sect. 2.4). Together, these two data streams allowed inference of whether users adapted to thermal stress by changing the timing, duration, or shade-location of their use of each site.

### Subjective thermal comfort survey

As the microclimatic monitoring was being conducted, questionnaires were administered. In total, 413 valid on-site questionnaires were collected at the selected sites. The number of questionnaires distributed at each site is summarized in Table 2. Respondent demographics were balanced across the two study areas, with female respondents constituting 57% and male respondents 43%; the dominant age group was 21–30 years (approximately 34%), followed by 31–40 years (approximately 24%). The questionnaire was designed in accordance with ISO 10,551 (2019) guidelines and the ASHRAE Standard 55 (2017). The questionnaire collected information regarding demographics, thermal comfort perceptions, clothing items (ISO 9920), pre-survey 10-minute activity level (ISO 8996), and environmental conditions, such as solar exposure, shading, and partial shading. Each response was timestamped and geolocated.

The primary components included thermal sensation vote (TSV) and thermal comfort vote (TCV), which were used to calculate mean TSV (MTSV) values across frequency intervals. TSV was assessed using a 9-point scale, as proposed by Potchter et al. (2022), with response options of very cold (− 4), cold (− 3), cool (− 2), slightly cool (− 1), neutral (0), slightly warm (1), warm (2), hot (3), and very hot (4). TCV was assessed using a 6-point symmetric scale, with exclusion of the neutral midpoint to enable clearer assessment of comfort perception. The response options were very uncomfortable (− 3), uncomfortable (− 2), slightly uncomfortable (− 1), slightly comfortable (1), comfortable (2), and very comfortable (3).

In addition, the questionnaire assessed respondents’ expectations regarding various thermal parameters, including temperature, solar radiation, wind speed, and humidity. Solar radiation expectations were elicited using 5 °C intervals of mean radiant temperature; wind expectations were elicited using the Beaufort scale (Level 0: <0.3 m/s, calm; Level 1: 0.3–1.5 m/s, light air; Level 2: 1.6–3.3 m/s, light breeze; Level 3: 3.4–5.4 m/s, gentle breeze; Central Weather Administration, Taiwan); humidity expectations were elicited using 5% relative-humidity intervals.

These were rated on a 3-point scale, with response options of prefer cooler (− 1), no change (0), and prefer warmer (+ 1). Overall thermal environment satisfaction was evaluated using a 7-point scale, as recommended by ASHRAE Standard 55 (2017), with endpoints ranging from − 3 (very dissatisfied) to + 3 (very satisfied). To enable contextualization of the thermal comfort responses, this study also collected respondents’ clothing insulation and metabolic rate via the questionnaire, with clothing insulation values estimated using the ISO 9920 (2007) standard insulation table and metabolic rate values estimated using the ISO 8996 (2004) standard activity table. The full questionnaire is provided as Online Resource 1.

### Thermal comfort assessment

This study adopted mPET as the primary indicator for assessing outdoor thermal comfort. mPET was developed to address the limitations of PET in hot and humid environments, particularly in terms of enhancing PET’s sensitivity to humidity and clothing parameters (Chen and Matzarakis 2018). According to Lin (2019), mPET is more effective than PET in humid environments and has been widely applied and validated in contemporary thermal comfort studies conducted in humid regions.

This study compiled and processed meteorological data—including air temperature (Ta, °C), relative humidity (Rh, %), globe temperature (Tg, °C), and wind speed (V, m/s)—and data on clothing insulation and metabolic rate (collected using the questionnaire). RayMan Pro was used to compute PET and mPET values (Matzarakis et al. 2007, 2010). To investigate thermal sensation and thermal comfort ranges at each study site, the relationships between mPET (X) and TSV (Y) values at each site were examined. Linear regression analysis was used to examine these relationships and calculate a theoretical neutral temperature. In addition, because both excessively low and excessively high mPET values can result in thermal discomfort, a second-degree polynomial regression analysis was conducted to examine the relationship between mPET (X) and TCV (Y) values to define the optimal thermal comfort range for the studied areas.

## Results

### Subjective thermal responses by site

**Fig. 2:**
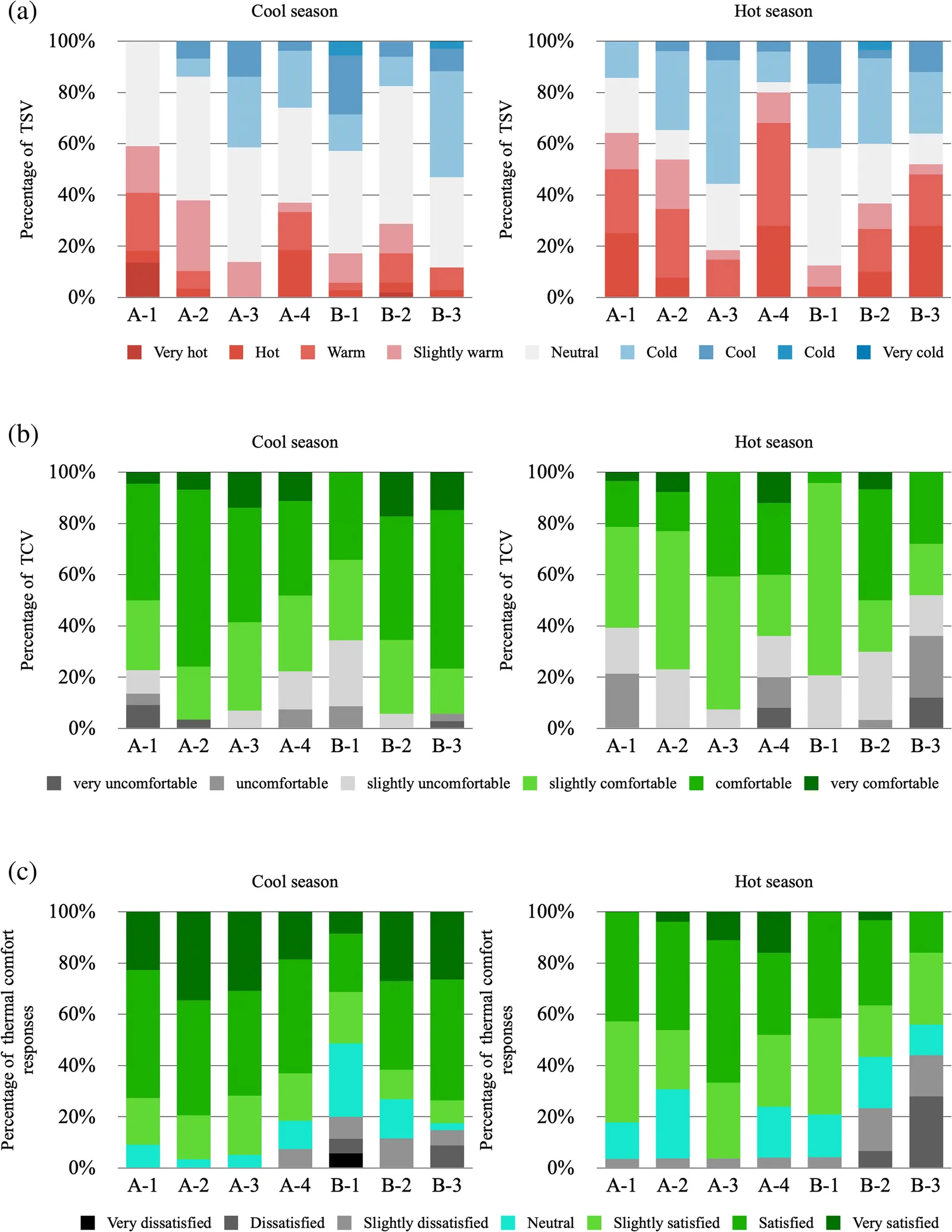
(**a**) Thermal sensation, (**b**) comfort level, and (**c**) thermal satisfaction across all measurement sites during cool and hot seasons

This section presents the distribution and average values with respect to thermal sensation, thermal comfort, and overall thermal satisfaction across all measurement sites during the cool and hot seasons (Fig. 2). The results reveal that variations in small-scale urban thermal environments significantly influence users’ thermal perceptions and comfort levels, with this being particularly true in the Zhongxing New Village sites.

In terms of thermal sensation, during the cool season, Site A-1 (a plaza space) was associated with a higher proportion of warm-related responses, with few reports of coldness. Mean TSV at this site was 1.3—the highest among all sites—indicating a general perception of warmth to heat. During the hot season, more than 60% of the respondents at most sites reported positive thermal sensations (i.e., warm, hot, or very hot). Notably, more than 80% of the respondents at Sites A-1 and A-4 expressed feelings of warmth. By contrast, only approximately 40% of the respondents at Site A-3 (a park space) reported warmth, likely because of the area’s extensive shading, which effectively reduced perceived heat.

Regarding thermal comfort, respondents at most sites expressed positive comfort during the cool season, indicating that outdoor environments were generally perceived to be comfortable. Site B-1 (a plaza space) was associated with a relatively high proportion of negative comfort responses (30%), and at Sites A-1 and A-4, more than 20% of the respondents reported feeling discomfort. At Site B-3 (a park space), the proportion of respondents’ reports of discomfort was significantly higher in the hot season than in the cool season, suggesting that the thermal conditions at this site were less favorable in summer. By comparison, the comfort level at Site A-3 was consistently high, with 90% of the respondents reporting thermal comfort, even in the hot season. This finding highlights the effectiveness of the shade incorporated into its design and its spatial configuration in enhancing outdoor comfort during high-temperature periods.

### Thermal expectations by intervals

**Fig. 3:**
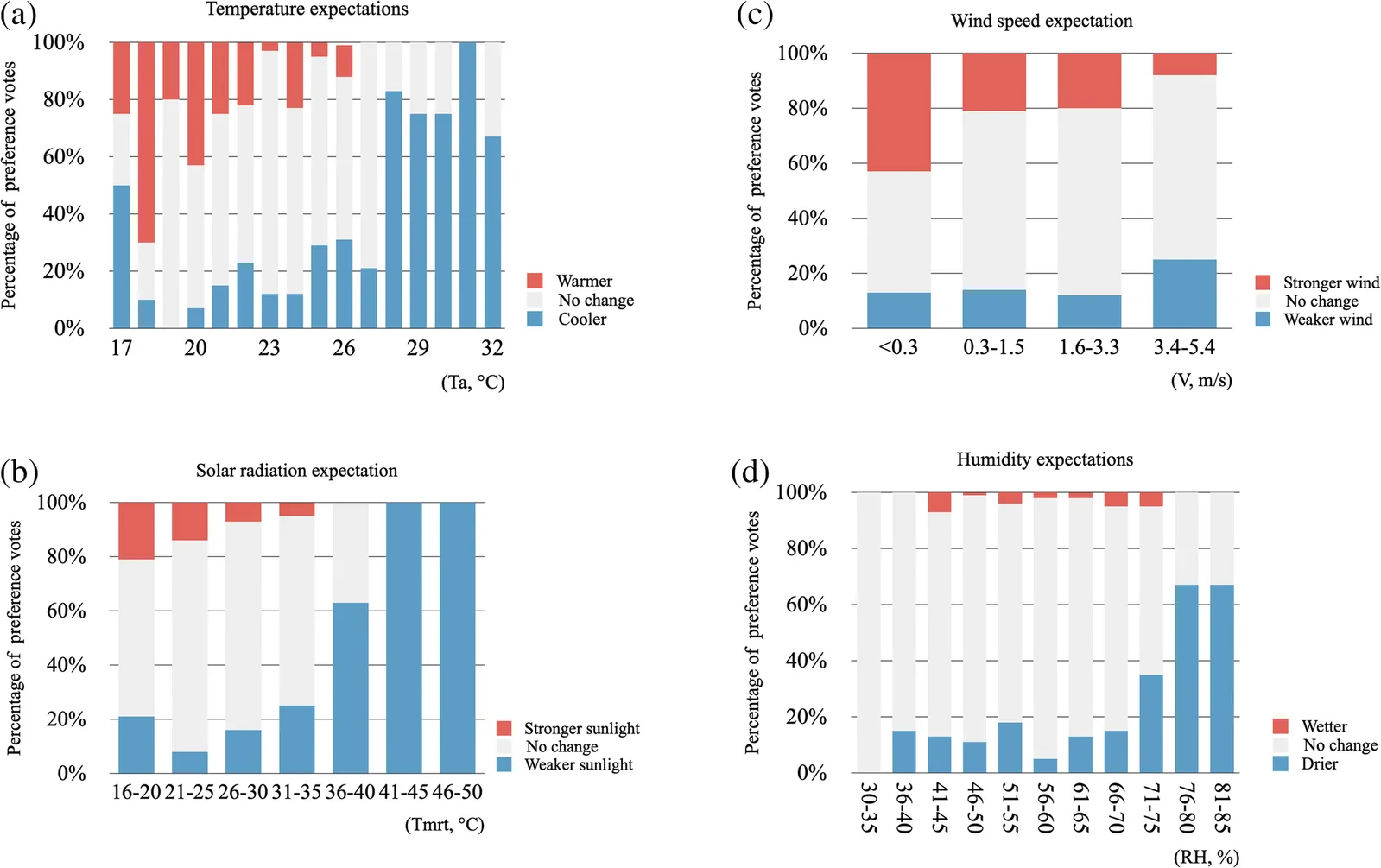
Thermal expectations of (**a**) temperature, (**b**) solar radiation, (**c**) wind speed, and (**d**) humidity

This study categorized thermal expectations in terms of temperature, solar radiation, wind speed, and humidity (Fig. 3). Respondents were asked to express their preferences under specific thermal conditions on a 3-point scale (prefer cooler − 1, no change 0, and prefer warmer + 1).

Regarding temperature expectations, more than 80% of the respondents reported that they preferred “no change” when the temperature was 23 °C, indicating that 23 °C is the most favorable and comfortable temperature. When temperatures exceeded 28 °C, more than 60% of the respondents reported that they preferred cooler conditions.

Regarding solar radiation expectations, more than 70% of the respondents were satisfied with the current solar conditions when the mean radiant temperature ranged from 16 to 30 °C. When the mean radiant temperature exceeded 36 °C, most respondents reported wishing for a reduction in solar intensity.

Regarding wind speed radiation expectations, more than 60% of the respondents reported wishing for no change when the wind speed was between 0.3 and 5.4 m/s (Beaufort scale Levels 1–3). At wind speeds below 0.3 m/s (Beaufort Level 0), respondents were significantly more likely to wish for higher wind speeds. These results indicate a general preference for mild airflow in outdoor settings.

Regarding humidity expectations, more than 80% of the respondents were satisfied with the current humidity conditions when humidity levels ranged from 30% to 70%. When humidity exceeded 70%, a higher number of respondents reported wishing for drier conditions, indicating that high humidity negatively influenced comfort.

### Relationship between subjective perception and thermal environment

To further understand how subjective thermal responses correlate with environmental conditions, this study analyzed the relationship between microclimatic parameters, mPET, and thermal perceptions during the cool and hot seasons. The microclimatic variables and mPET values were interval variables, and the TSV and TCV values were ordinal variables. Therefore, Spearman’s rank correlation coefficient was employed for analysis.

As listed in Table 3, air temperature, mean radiant temperature, and mPET were all positively and significantly correlated with TSV (thermal sensation) in both the cool and hot seasons. This indicates that higher values for these climate variables corresponded with the perception of higher heat. By contrast, humidity was negatively correlated with TSV. Because the highest humidity values in this dataset tended to occur on cooler, overcast occasions, this most likely reflects the inverse covariation between humidity and air temperature rather than an independent effect of humidity on thermal sensation. In terms of thermal comfort (TCV), the results indicated that during the cool season, air temperature and mean radiant temperature had a significant but weak positive correlation with TCV. During the hot season, air temperature, mean radiant temperature, and mPET exhibited a significant negative correlation with TCV, indicating that lower temperatures were associated with higher comfort levels.

Overall, the analysis demonstrates that air temperature, mean radiant temperature, and mPET consistently influence thermal sensation across seasons. In particular, during the hot season, these parameters are critical factors affecting outdoor thermal comfort. By contrast, during the cool season, the relationship between microclimatic parameters and thermal comfort was generally weaker.

**Table 3:** Correlation Between microclimatic parameters, mPET and thermal perception

Season	microclimatic parameters	Thermal Sensation	Thermal Comfort
**Cool Season**	Air Temperature	0.438**	-0.174**
	Relative Humidity	-0.285**	-0.005
	Wind Speed	-0.066	-0.120
	Mean Radiant Temperature	0.398**	-0.180**
	mPET	0.449**	-0.093
**Hot Season**	Air Temperature	0.407**	-0.329**
	Relative Humidity	-0.234**	0.200**
	Wind Speed	0.130	-0.028
	Mean Radiant Temperature	0.446**	-0.275**
	mPET	0.373**	-0.218**

^a^ The values in the table represent Spearman’s rank correlation coefficients (rs)

^b^ *p < 0.05, **p < 0.01

^c^ | rs = 0.8 ~ 1 | = Very High Correlation, | rs = 0.6 ~ 0.79 | = High Correlation, | rs = 0.4 ~ 0.59 | = Moderate Correlation, | rs = 0.2 ~ 0.39 | = Low Correlation, | rs = 0.01 ~ 0.19 | = Very Low Correlation

### Behavioral patterns of outdoor space use

This study also considered clothing insulation and metabolic rates as key behavioral indicators of thermal adaptation and space use. During the cool season, the mean clothing insulation across the sites ranged from 0.64 to 0.82 clo, with an overall mean of 0.70 clo. During the hot season, the mean clothing insulation ranged from 0.42 to 0.56 clo, with an overall mean of 0.49 clo (Table 4). In contrast to Lin et al. (2011), who observed average values of 0.71 clo in the cool season and 0.60 clo in the hot season in central Taiwan, the current study observed lower in summer clothing insulation.

The participants’ primary activities within the 10 min prior to the survey were walking and resting. At Site A-1, a transitional plaza, the participants had predominantly been walking prior to completing the survey, whereas at Site A-2, which is mostly used for family-oriented activities, the participants had primarily been sitting or resting. Sites A-1 and A-2 illustrate the two extremes of this activity range; the full distribution for all seven sites is given in Table 4 and generally follows each site’s spatial typology, with transitional plazas being walking-dominated and play or rest areas being sitting-dominated. These activity patterns broadly correspond to the intended function of each space and may in turn influence metabolic rate and perceived comfort.

**Table 4:** Clothing insulation and metabolic rate at each site during cool and hot seasons

		A-1	A-2	A-3	A-4	B-1	B-2	B-3	Mean
**Cool season** **(clo)**	Min	0.25	0.39	0.43	0.32	0.44	0.23	0.31	0.34
	Max	1.37	1.19	1.15	1.29	1.74	1.31	1.46	1.36
	Mean	0.66	0.66	0.64	0.67	0.78	0.68	0.82	0.70
	S.D.	0.23	0.17	0.16	0.23	0.29	0.25	0.29	0.23
**Hot season** **(clo)**	Min	0.23	0.23	0.21	0.23	0.23	0.21	0.25	0.23
	Max	0.72	0.72	0.74	0.92	0.72	0.68	0.90	0.77
	Mean	0.50	0.52	0.45	0.45	0.50	0.42	0.56	0.49
	S.D.	0.14	0.17	0.16	0.17	0.13	0.14	0.16	0.15
**Cool season** **(%)**	Lying down	0	0	0	0	0	0	0	—
	Sitting (resting)	22.7	44.8	17.2	14.8	48.6	9.6	23.5	—
	Sedentary activity	9.1	10.3	6.9	14.8	14.3	0	2.9	—
	Standing / Light activity	4.5	20.7	13.8	14.8	2.9	7.7	8.8	—
	Walking	45.5	20.7	58.6	48.1	34.3	65.4	47.1	—
	Standing / Moderate activity	4.5	0	0	3.7	0	17.3	2.9	—
	Exercising	13.6	3.4	3.4	3.7	0	0	14.7	—
**Hot season** **(%)**	Lying down	0	0	0	0	0	0	0	—
	Sitting (resting)	7.1	53.8	37	12	29.2	20	40	—
	Sedentary activity	25	15.4	3.7	8	0	0	8	—
	Standing / Light activity	14.3	15.4	22.2	36	4.2	26.7	12	—
	Walking	50	11.5	25.9	36	66.7	40	28	—
	Standing / Moderate activity	0	0	3.7	0	0	0	0	—
	Exercising	3.6	3.8	7.4	8	0	13.3	12	—

### Correlation between spatial use and thermal conditions

This section presents an exploration of the relationship between spatial use and thermal conditions, with a specific focus on mPET values. Linear regression analyses were conducted to examine the relationship between mPET and user counts across different sites (Table 5). As the seven sites were surveyed on separate days rather than simultaneously, the associations reported below reflect both spatial differences between sites and day-to-day variation in weather; the meteorological conditions for each measurement day are given in Table 2.

In open multifunctional plazas (A-1, A-4, and B-3), in both the cool and hot seasons, significant negative correlations were noted between mPET values and user counts—higher mPET values were correlated with lower user counts. In children’s play areas (A-2 and B-2), time of day appeared to have a greater influence on user count than did mPET. In heavily shaded areas, such as A-3, a positive correlation was observed between mPET values and user counts during the cool season, possibly because of lower temperatures due to dense shading in these areas, rendering slightly warmer conditions more desirable. By contrast, for single-function spaces, such as B-1, no clear correlation was noted between user count and mPET, indicating limited behavioral flexibility in such spaces.

Overall, at most of the sites, a negative correlation was noted between mPET and user count across both seasons; this finding differs from those reported in the literature (Lin 2009; Lin et al. 2013), which indicated positive correlations in the cool season and negative correlations in the hot season. In the present study, some cool-season mPET values exceeded 30 °C, suggesting elevated temperature conditions that may explain the consistent negative correlations. Only sites with high shading (e.g., tree canopies) were associated with positive correlations in the cool season, with shading improving comfort despite the elevated temperatures and leading to increased usage.

In the hot season, user counts were higher in parks and play areas than in other areas despite their relatively high temperatures, likely because users could move between shaded and nonshaded zones. Conversely, in single-function spaces such as plazas, limited behavioral options led to consistently lower usage regardless of thermal conditions. These findings align with those of Deng et al. (2022), highlighting that spatial use behaviors are the result of complex interactions among multiple factors. Notably, thermal conditions are influential but not the sole determinant of outdoor space utilization.

**Table 5:** Relationship Between Microclimate Variables, mPET, and User Counts at Each Site During Cool and Hot Seasons. Tabulated values are Spearman’s rank correlation coefficients between each variable and user count; mPET(R²) is the coefficient of determination from the linear regression of user count on mPET

		A-1	A-2	A-3	A-4	B-1	B-2	B-3
**User Count (Cool Season)**	Air Temperature	-0.800**	-0.321	0.585*	-0.513*	-0.487	-0.742**	-0.773**
	Relative Humidity	0.756**	-0.038	-0.694**	0.210	0.629**	0.733**	0.734**
	Wind Speed	-0.281	0.098	-0.320	0.230	0.183	0.363	-0.513*
	Mean Radiant Temperature	-0.753**	-0.783**	0.424	-0.757**	-0.503*	-0.812**	-0.565*
	mPET	-0.783**	-0.762**	0.605*	-0.622*	-0.456	-0.756**	-0.702**
	mPET(R²)	0.61	0.58	0.36	0.38	0.21	0.57	0.49
**User Count (Hot Season)**	Air Temperature	-0.854**	0.270	0.104	-0.447	-0.045	0.757**	-0.930**
	Relative Humidity	0.616*	-0.750**	-0.757**	0.458	0.354	-0.626**	0.047
	Wind Speed	-0.579*	0.027	0.179	0.197	-0.090	-0.742**	-0.451
	Mean Radiant Temperature	-0.809**	-0.548*	-0.361	-0.665**	-0.116	0.841**	-0.928**
	mPET	-0.764**	-0.214	-0.267	-0.708**	0.007	0.784**	-0.904**
	mPET(R²)	0.58	0.04	0.074	0.49	—	0.61	0.82

^a^ The values in the table represent Spearman’s rank correlation coefficients (rs)

^b^ *p < 0.05, **p < 0.01

^c^ | rs = 0.8 ~ 1 | = Very High Correlation, | rs = 0.6 ~ 0.79 | = High Correlation, | rs = 0.4 ~ 0.59 | = Moderate Correlation, | rs = 0.2 ~ 0.39 | = Low Correlation, | rs = 0.01 ~ 0.19 | = Very Low Correlation

### Outdoor thermal comfort benchmarks

**Fig. 4:**
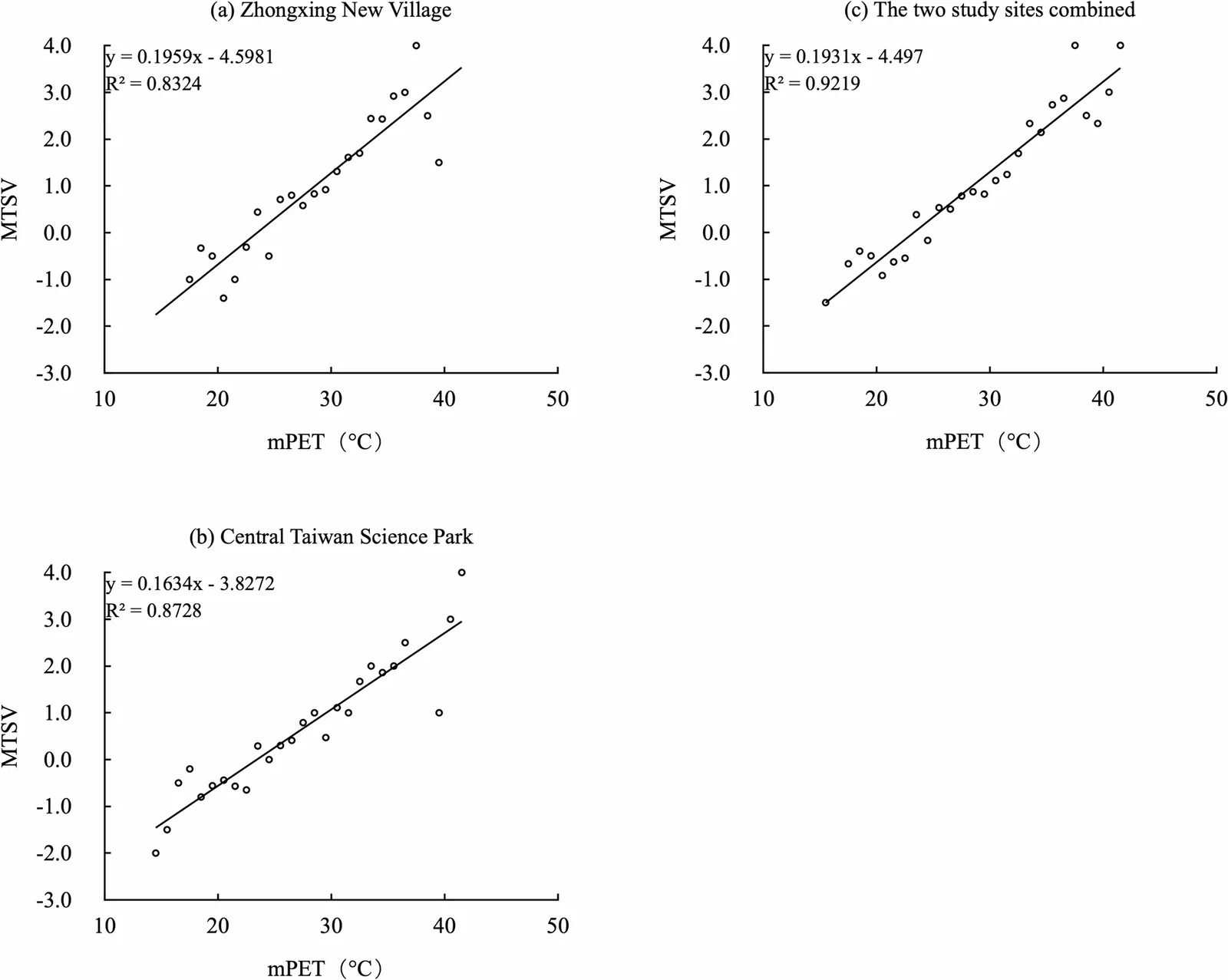
Relationship between thermal sensation and mPET in (**a**) Zhongxing New Village, (**b**) Central Taiwan Science Park, and (**c**) the two study sites combined

This section presents a further examination of the linear relationship between thermal sensation and mPET to establish a baseline for a neutral temperature in Cwa climate zones. A linear regression was performed in which MTSV was used as the dependent variable and mPET was used as the independent variable (Fig. 4), with the following equation:

*MTSV=0.1931× mPET-4.4975*

The model yielded a coefficient of determination (R²) of 0.9219, indicating that 92.19% of the variance in MTSV could be explained by mPET. The result was significant (*p* < 0.001), confirming that a strong relationship existed between mPET and thermal sensation. When MTSV = 0 (representing thermal neutrality) was substituted into the equation, the annual outdoor neutral temperature was calculated to be 23.3 °C mPET, with this representing the point at which individuals feel neither hot nor cold, in line with definitions provided by Nikolopoulou and Lykoudis (2006) and Lin (2009).

To enhance the regional applicability of the findings, separate analyses were conducted for the two study areas. Zhongxing New Village had a neutral temperature of 23.47 °C mPET, and the Central Taiwan Science Park had a neutral temperature of 23.42 °C mPET. The two values are closely comparable, differing by only 0.05 °C mPET.

**Fig. 5:**
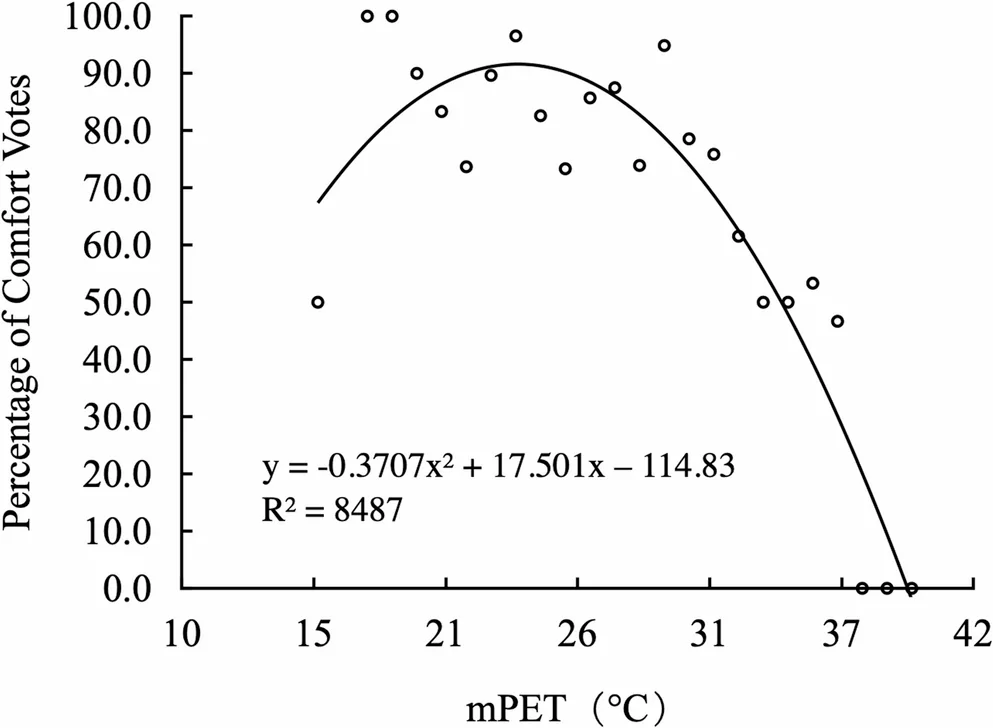
Relationship between thermal comfort percentage and mPET in Taiwan’s Cwa climate zone

To determine the annual acceptable thermal comfort range, this study used TCV, obtaining responses that were placed into two categories: comfortable (including scores of + 1, +2, and + 3) and uncomfortable (including scores of − 3, −2, and − 1). Thermal comfort percentages were calculated across different mPET intervals. According to ASHRAE Standard 55 (2017), acceptable comfort conditions should be experienced by at least 90% of users; the proportion of users experiencing discomfort should remain below 10%. Because both high and low mPET levels can cause discomfort, a quadratic regression analysis was applied to evaluate the relationship between mPET and comfort percentage. As presented in Fig. 5, the model yielded an R² of 0.8487 (*p* < 0.001), indicating a strong effect of mPET on thermal comfort. The regression equation is as follows:

$$\:Thermal\:Comfort\:Percentage=-0.3707\:\times\:\:\left(mPET\right)^2\:+\:17.501\:\times\:\:mPET-114.83$$

According to this model, mPET values corresponding to 90% comfort indicate the annual acceptable thermal comfort range. The results indicated a range of 21.4 to 25.8 °C mPET, which represents the thermal environment conditions perceived to be comfortable by most users. The results of this analysis provide an evidence-based thermal comfort benchmark specific to Taiwan’s Cwa climate zone.

## Discussion

### Outdoor thermal comfort design strategies

This study revealed a strong correlation between an outdoor environment’s thermal conditions and spatial usage, with a focus on Taiwan’s Cwa climate zone, where the hot season lasts as long as 9 months. The findings of the study indicate that plaza-type spaces are highly susceptible to thermal influences and that usage in such spaces is significantly affected by temperature during both cool and hot seasons. In highly shaded environments such as Site A-3, user counts increased with air temperature and mPET during the cool season. This pattern is consistent with the radiative basis of outdoor heat exchange: shading lowers thermal load primarily by intercepting shortwave radiation and reducing the mean radiant temperature (Tmrt) rather than air temperature (Du et al. 2020; Lam et al. 2023), so that the dense canopy improving hot-season comfort leaves the space radiatively cool, and less used, in winter. In function-specific spaces such as playgrounds and sports fields, user numbers were relatively insensitive to hot-season conditions; consistent with recent attendance studies in humid subtropical parks (Nikolopoulou and Lykoudis 2006; Nikolopoulou and Steemers 2003), this suggests that attendance there is driven mainly by the spaces’ dedicated function and social or recreational needs, with thermal comfort acting alongside rather than independently of those purposes.

These shade-seeking and time-shifting behaviors are themselves forms of behavioral adaptation, while psychological adaptation—expectation, experience, perceived control—further shapes the subjective response (Li et al. 2024; Rao et al. 2024; Deng et al. 2022), which together explain the low-to-moderate microclimate–vote correlations (Table 3). Accordingly, outdoor space design should account for seasonal variability: adjustable or strategically placed shading along plaza edges can mitigate hot-season heat stress, while in necessary-use spaces such as playgrounds, adaptive shading lets users self-select the thermal environment that suits them.

Overall, the mechanisms governing thermal response here, including radiative shading, behavioral and psychological adaptation, and function-driven use, align with the broader outdoor thermal comfort literature, supporting the soundness of the approach; what distinguishes the Cwa urban context is not these mechanisms but the resulting calibration, since the neutral temperature and PET thresholds derived here differ systematically from those of other climate zones (Sect. 4.3), precisely why a locally calibrated scale, rather than a transferred one, is needed for Cwa urban environments.

### Thermal comfort across urban sites at the same latitude

This study focused on two urban locations within Taiwan’s Cwa climate zone. The outdoor neutral temperatures calculated in this study were 23.47 °C mPET and 23.42 °C mPET in Zhongxing New Village and the Central Taiwan Science Park, respectively. The annual acceptable thermal comfort range, defined as the mPET interval in which at least 90% of respondents reported thermal comfort, was determined to be 21.4 to 25.8 °C mPET for both sites.

The results of this study are consistent with those of Lin’s (2009) study, which was conducted at the National Taiwan Museum of Fine Arts in Taichung, an area that also has a Cwa climate and is at a similar latitude as the study areas in the present study. Lin (2009) reported a neutral temperature of 23 °C mPET and a 90% comfort acceptance range of 21.3 to 28.5 °C PET. The close agreement in neutral temperature between the present sites and Lin (2009) lends further confidence to the derived Cwa values and is consistent with a degree of within-climate-zone consistency in thermal comfort benchmarks.

### Comparison of thermal comfort and PET scales across climate zones

Building on the neutral temperature (23.3 °C mPET) and the 90% acceptable comfort range (21.4–25.8 °C mPET) derived above, a PET threshold classification for the two study sites was constructed for comparison with reference scales from other climate zones. Following the method of Matzarakis and Mayer (1996), eight transition thresholds were defined on the 9-point thermal sensation scale (3.5, 2.5, 1.5, 0.5, − 0.5, − 1.5, − 2.5, and − 3.5). The neutral range (approximately 22–26 °C) was rounded to integer values to facilitate application, and for the extreme sensation levels (− 2.5 and − 3.5), where sample sizes were limited, values were estimated by linear regression (Lai et al. 2014). The resulting categories are summarized in Table 6 alongside PET scales reported for other climate zones, including Sun Moon Lake in Taiwan (Cfa), the Schwarzwald in Germany (Dfc), Tianjin in China (Dwa), and Chandigarh in India (Cwg).

Thermal comfort conditions vary substantially across climate zones. Several studies have highlighted considerable differences in outdoor neutral temperatures across regions. For example, for Sun Moon Lake, which has a Cfa climate, a neutral temperature of 27.2 °C PET was recorded (Lin and Matzarakis 2008); that in Chandigarh in India (Cwg climate) was reported to be 24.09 °C PET (Manavvi and Rajasekar 2022); that in Tianjin in China (Dwa climate) was reported to be 20.5 °C PET (Lai et al. 2014); and that in Changsha in China (Cfa climate) was reported to be 26.2 °C PET (Deng et al. 2022). These differences underscore how climatic conditions directly influence subjective thermal perception and the range of thermal neutrality.

A comparison of PET-based thermal comfort scales across different climate zones is summarized in Table 6. The results reveal substantial variation in PET classification thresholds under distinct climatic conditions. The PET thresholds derived for the present Cwa study sites are distinct from those reported for other Köppen–Geiger climate zones, which supports the need for climate-zone-specific thermal perception scales rather than the application of a single nationwide PET classification. Even within the same country, noticeable differences emerge. For example, this study, which involved a Cwa climate zone, and the study by Lin and Matzarakis (2008), which involved a Cfa climate zone, yielded significantly different PET thresholds. Although the sites in these studies were all located in central Taiwan at similar latitudes, differences in altitude (a difference of nearly 600 m) and climate classification were associated with notably different thermal comfort outcomes.

To clarify how site geography relates to thermal neutrality, the latitude and altitude of each comparison site are now reported in Table 6. Across these sites, neutral PET does not follow a simple monotonic trend with either variable. The highest-altitude site (Sun Moon Lake, 748 m) shows the highest neutral temperature (27.2 °C PET)—the opposite of a straightforward cooling-with-altitude expectation—whereas the highest-latitude, near-sea-level site (Tianjin, ~ 39° N, ~ 5 m) shows one of the lowest (20.5 °C PET), and the inland subtropical site at intermediate latitude (Chandigarh, 30.7° N, 321 m) likewise does not fall on a consistent gradient (24.09 °C PET). These contrasts indicate that latitude and altitude act only as partial and mutually confounded proxies; neutral temperature appears instead to track each location’s local background thermal climate and the resulting thermal adaptation and expectations of its users (Cheung and Jim 2018; Lin and Matzarakis 2008). The clearest signal is the within-Taiwan comparison: the present Cwa sites and Sun Moon Lake lie at almost the same latitude (~ 24° N) but differ by nearly 600 m in altitude and by one Köppen sub-class, a difference associated with a neutral-temperature gap of about 4 °C. At comparable latitude, therefore, altitude-linked differences in background climate—rather than latitude itself—are associated with markedly different thermal neutrality. Because the comparison sites differ not only in geography but also in measurement index (PET versus the mPET used here), survey period, and population characteristics, these relationships are interpreted qualitatively rather than as a formal statistical model; nonetheless, they consistently reinforce the inadequacy of applying a single nationwide PET classification.

The study areas investigated in this study are primarily situated in the urbanized western region of Taiwan and are key population and activity centers. Accordingly, the PET thermal comfort scale derived here is intended as a localized reference for climate-responsive thermal comfort evaluation in Taiwan’s major Cwa metropolitan areas, complementing rather than replacing existing national references.

**Table 6:** Summary of PET scales from this study and previous studies in different climate zones

Thermal sensation	This study, Taiwan (Cwa)	Sun Moon Lake, Taiwan ^a^ (Cfa)	Schwarzwald, Germany ^b^ (Dfc)	Tianjin, China ^c^ (Dwa)	Chandigarh, India ^d^ (Cwg)
Latitude	~ 24.0° N	23°52′ N	~ 48° N	~ 39.1° N	30°44′ N
Altitude (m a.s.l.)	~ 100–200	748	— ᵉ	~ 5	321
Neutral temperature (°C)	23.3 ^**f**^	27.2	— ᵉ	20.5	24.09
Very cold					
	6*	14	4	-16*	—
Cold					
	11*	18	8	-11*	—
Cool					
	16	22	13	-6*	7.4
Slightly cool					
	22	26	18	11	18.7
Neutral					
	26	30	23	24	29.7
Slightly warm					
	30	34	29	31	37.1
Warm					
	35	38	35	36*	46.1
Hot					
	40	42	41	46*	53.8
Very hot					

* Sensation obtained by linear regression.

^a^ Source: Lin and Matzarakis (2008).

^b^ Source: Matzarakis and Mayer (1996).

^c^ Source: Lai et al. (2014)

^d^ Source: Manavvi and Rajasekar (2022).

^e^ The Schwarzwald column represents a Central-European regional PET reference scale, not a single field site.

ᶠ The present study reports mPET; the comparison sites report PET.

Several limitations should be considered when interpreting these findings. First, mPET was adopted specifically because it improves on PET’s known weak sensitivity to humidity and clothing insulation (Chen and Matzarakis 2018; Lin et al. 2019); nonetheless, both indices are steady-state, whole-body energy-balance models that do not represent the transient, non-uniform exposure of real outdoor use, and the mean radiant temperature on which they depend is itself subject to appreciable estimation uncertainty (Kántor and Unger 2011). The thresholds reported here should therefore be read as index-specific reference values rather than absolute comfort limits.

Second, as noted in Sect. 3.5, the seven sites were surveyed on separate days rather than simultaneously, so the site-level associations partly confound genuine spatial differences with day-to-day weather; the per-day conditions are reported in Table 2 to allow readers to weigh this, but the two cannot be fully separated in the present design. Relatedly, and as reflected in Table 3, the negative correlation between relative humidity and thermal sensation runs counter to the usual expectation that higher humidity intensifies heat perception (Höppe 1999); because the highest humidity coincided with cooler, overcast conditions, this most likely reflects humidity–temperature covariation rather than an independent humidity effect, which only a larger multivariate analysis could isolate.

Finally, the present thresholds were not linked to specific physical design parameters such as sky view factor, tree density, or shading geometry, and the study spans two Cwa sites over a single annual cycle. Establishing direct, evidence-based links between physical environmental features and thermal response across a broader set of Cwa urban sites is an important direction for future work and a prerequisite before the scale is used to inform design rather than evaluation.

## Conclusion

This study conducted in-situ microclimatic measurements and a thermal comfort survey at two urban sites in a humid subtropical area of Taiwan with a Cwa climate. By integrating the mPET index with subjective user feedback, the research established a localized and adaptive thermal comfort evaluation framework. The findings reveal a strong linear relationship between mPET and thermal sensation, and the study calculated a neutral temperature of 23.3 °C mPET for the study areas. Furthermore, the thermally acceptable comfort range for 90% of respondents was identified as 21.4–25.8 °C mPET. On the basis of these results, this study developed a context-specific PET thermal sensation scale for Cwa climate zones. Comparison with PET scales reported for other climate zones (Table 6) indicated that these thresholds differ from those calibrated elsewhere, consistent with the regional specificity of outdoor thermal perception; because these differences were not formally tested for statistical significance, they are interpreted as indicative rather than confirmatory. The analysis of spatial usage further showed that thermal conditions influenced attendance in open plaza-type spaces more strongly than in function-specific spaces, indicating that thermal comfort is one of several factors shaping outdoor space use.

The PET scale derived here is intended as a localized reference for thermal comfort evaluation in Taiwan’s Cwa urban areas, complementing rather than replacing existing national references. Future studies could expand on the scope of this study to include other climates (e.g., Cfa and Am climates) and incorporate behavioral preferences, psychological expectations, and sociocultural factors to achieve a more comprehensive and contextual understanding of outdoor thermal comfort. Establishing direct links between specific physical features, such as shading geometry, sky view factor, or tree density, and thermal response across a wider set of Cwa sites remains an important direction for future work.

## Supplementary information

Below is the link to the electronic supplementary material.

Supplementary Material 1 (PDF 91 KB)

## Data Availability

Not applicable.
