# Supplementary material for: Limitations of employing a unified physiological equivalent temperature scale for assessing thermal comfort in Taiwan’s Cwa urban climate
Source: Int J Biometeorol. 2026 Aug 20;70(9):240. doi: 10.1007/s00484-026-03294-2 (PMC13493517; doi:10.1007/s00484-026-03294-2)
Supplement: Supplementary file 1 [file 484_2026_3294_MOESM1_ESM.pdf]

Outdoor Thermal Comfort Subjective Questionnaire

Part I. Perception of Outdoor Thermal Comfort

1. How do you perceive the thermal sensation (hot/cold) of the outdoor environment at your current location? (Single choice)

☐ Very cold (–4) ☐ Cold (–3) ☐ Cool (–2) ☐ Slightly cool (–1) ☐ Neutral (0) ☐ Slightly warm (1) ☐ Warm (2) ☐ Hot (3) ☐ Very hot (4)

2. How comfortable do you feel in the outdoor environment at your current location? (Single choice)

☐ Very uncomfortable (–3) ☐ Uncomfortable (–2) ☐ Slightly uncomfortable (–1) ☐ Slightly comfortable (1) ☐ Comfortable (2) ☐ Very comfortable (3)

3. What is your expectation regarding the air temperature at your current location? (Single choice)

☐ Prefer cooler (–1) ☐ No change (0) ☐ Prefer warmer (1)

4. What is your expectation regarding the sunlight at your current location? (Single choice)

☐ Prefer weaker sunlight (–1) ☐ No change (0) ☐ Prefer stronger sunlight (1)

5. What is your expectation regarding the air movement (wind) at your current location? (Single choice)

☐ Prefer less wind (–1) ☐ No change (0) ☐ Prefer more wind (1)

6. What is your expectation regarding the humidity at your current location? (Single choice)

☐ Prefer drier (–1) ☐ No change (0) ☐ Prefer more humid (1)

7. What is your overall satisfaction with the thermal environment of the entire Central Taiwan Science Park / Zhongxing New Village? (Single choice)

Very dissatisfied ☐ –3 ☐ –2 ☐ –1 ☐ 0 ☐ 1 ☐ 2 ☐ 3 Very satisfied

Part II. Respondent Background Information

1. Sex:

☐ Male ☐ Female

2. Age:

☐ 11–20 ☐ 21–30 ☐ 31–40 ☐ 41–50 ☐ 51–60 ☐ ≥ 61

Part III. Human Factors of Outdoor Thermal Comfort

1. What was your main activity during the 10 minutes before completing this questionnaire? (Single choice)

☐ Lying down ☐ Sitting (resting) ☐ Sedentary activity ☐ Walking ☐ Exercising

☐ Standing / Light activity (e.g., chatting, looking after children, waiting)

☐ Standing / Moderate activity (e.g., working, cleaning, cooking) ☐ Other: ____________

2. Please check the boxes below according to your current clothing. (Multiple choices allowed)

(This item may be completed by the respondent or recorded by the surveyor based on observation.)

**Underwear / Next-to-skin**	☐ None ☐ Fitted vest (tank top) ☐ Short-sleeve undershirt ☐ Long-sleeve thermal undershirt ☐ Other: ______
**Upper body**	☐ None ☐ Sleeveless top ☐ Short-sleeve top / shirt ☐ Thin long-sleeve top / shirt ☐ Regular long-sleeve top / shirt ☐ Thick long-sleeve top / shirt ☐ Thin knit ☐ Thick sweater ☐ Knit vest ☐ Other: ______
**Jacket / Outerwear**	☐ None ☐ Light jacket ☐ Regular jacket ☐ Warm / heavy coat ☐ Down jacket ☐ Knit cardigan ☐ Gilet (vest) ☐ Other: ______
**Lower body**	☐ None ☐ Shorts ☐ Thin long trousers ☐ Regular long trousers ☐ Thick long trousers (fleece-lined) ☐ Thin skirt ☐ Thick skirt ☐ Other: ______
**One-piece garment**	☐ None ☐ Summer dress ☐ Winter dress ☐ Coveralls (work jumpsuit) ☐ Other: ______
**Socks**	☐ None ☐ Ankle socks ☐ Long socks ☐ Tights / stockings ☐ Other: ______
**Shoes**	☐ None ☐ Sandals / slippers ☐ Regular cloth / leather shoes ☐ Boots ☐ Other: ______

— End of questionnaire. Thank you sincerely for your assistance. —

To be completed by the surveyor

Location: ______________ Date: ____________ Time: ____________

Instrument sun-exposure status: ☐ Full shade ☐ Partial shade ☐ Full sun

Respondent sun-exposure status: ☐ Full shade ☐ Partial shade ☐ Full sun
